# Fatal AA-like bone marrow failure and invasive pulmonary aspergillosis after long-term pembrolizumab in squamous NSCLC: a case report

**DOI:** 10.3389/fimmu.2026.1800904

**Published:** 2026-04-23

**Authors:** Yawen Wang, Weigang Dong, Jianwen Qin

**Affiliations:** 1 Department of Respiratory and Critical Care Medicine, Chest Hospital, Tianjin University, Tianjin, China; 2 Department of Respiratory and Critical Care Medicine, Tianjin Chest Hospital, Tianjin, China

**Keywords:** aplastic anemia, immune checkpoint inhibitors, invasive pulmonary aspergillosis, non-small cell lung cancer, pembrolizumab

## Abstract

Immune checkpoint inhibitors (ICIs) such as pembrolizumab have substantially improved outcomes in advanced non–small cell lung cancer (NSCLC), including squamous histology, but prolonged exposure may be complicated by immune-related adverse events (irAEs) and opportunistic infections. We report a 58-year-old man with advanced squamous NSCLC who achieved durable tumor control after six cycles of pembrolizumab plus platinum-based chemotherapy, followed by pembrolizumab maintenance monotherapy (18 cycles). During the later course, he developed severe bacterial pneumonia, invasive pulmonary aspergillosis (IPA), and subsequent aplastic anemia (AA)–like bone marrow failure. Despite systemic antifungal therapy and supportive measures, he experienced progressive pancytopenia complicated by massive hemoptysis and ultimately died. This case underscores the dual nature of ICIs: while providing meaningful and sustained antitumor benefit, they may rarely precipitate life-threatening hematologic toxicity and facilitate severe opportunistic infections in a complex immunologic milieu. Close surveillance of blood counts and infectious complications is warranted during long-term ICI therapy; unexplained cytopenias or new/worsening radiologic abnormalities should prompt early bone marrow evaluation and comprehensive microbiologic work-up. Metagenomic next-generation sequencing (mNGS) may offer useful adjunctive evidence in diagnostically challenging infections, particularly when invasive sampling is not feasible, but results should be interpreted in conjunction with clinical and radiologic context within a multidisciplinary framework.

## 1 Introduction

Lung cancer remains a leading cause of cancer-related mortality worldwide, and outcomes for advanced non–small cell lung cancer (NSCLC) are generally poor (1). For advanced squamous NSCLC, the benefit of conventional platinum-based chemotherapy is limited, whereas immune checkpoint inhibitors (ICIs), particularly programmed cell death protein 1/programmed cell death ligand 1 (PD-1/PD-L1) inhibitors, have improved survival and are now part of standard first-line treatment strategies, typically in combination with chemotherapy (2–4). However, with broader use and longer treatment exposure, immune-related adverse events (irAEs) and related clinical challenges have garnered increasing attention (5, 6).

IrAEs can involve virtually any organ system, most commonly affecting the gastrointestinal tract, skin, liver, endocrine organs, and lungs (6). In contrast, hematologic irAEs (Hem-irAEs) are uncommon, with an estimated overall incidence of approximately 0.04%–3.6% (7). Among these, ICI-induced aplastic anemia (AA) or AA-like bone marrow failure is exceedingly rare and has been described largely in sporadic case reports (8). Its onset is unpredictable, occurring early after treatment initiation or as a delayed event months later (9, 10). Once established, AA-like marrow failure may progress rapidly and can be fatal, representing one of the most severe immune-mediated toxicities.

Opportunistic infections in the setting of ICI therapy have also emerged as an important clinical concern. ICIs are not considered classic immunosuppressive agents, and the overall risk of severe opportunistic infection with ICI monotherapy appears relatively low (11). Nevertheless, the risk of serious infections—including invasive pulmonary aspergillosis (IPA)—rises substantially in patients requiring high-dose corticosteroids and/or other immunosuppressants for irAE management and possibly with prolonged ICI exposure (12–14). Sporadic cases of IPA during PD-1/PD-L1 inhibitor therapy have been reported, often in patients without persistent neutropenia, making recognition difficult and outcomes frequently unfavorable (13–15).

To date, ICI-associated IPA and AA-like marrow failure have largely been reported as isolated events. The concurrence of these two rare and potentially lethal complications in a patient with squamous NSCLC who achieved sustained clinical benefit during long-term pembrolizumab therapy has rarely been reported. Here, we report this unusual fatal case to highlight the need for ongoing vigilance, timely diagnostic work-up, and careful risk–benefit reassessment in long-term ICI responders.

## 2 Case presentation

### 2.1 Initial presentation and oncologic treatment

**Figure 1:**
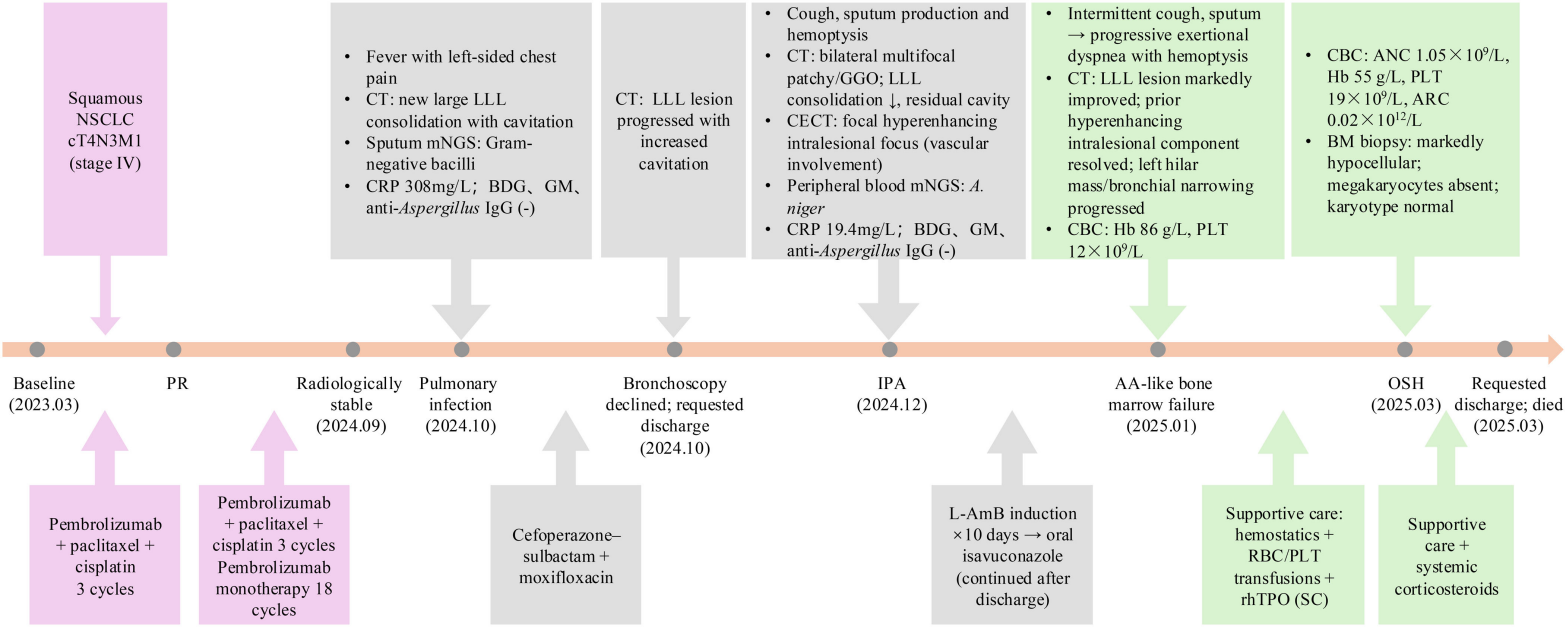
Timeline of events in this case. NSCLC, Non–small cell lung cancer; LLL, Left lower lobe; CT, Computed tomography; GGO, Ground-glass opacities; CECT, Contrast-enhanced computed tomography; mNGS, Metagenomic next-generation sequencing; A. niger, Aspergillus niger; CRP, C-reactive protein; BDG, Serum (1,3)-β-D-glucan assay; GM, Serum galactomannan assay; IPA, invasive pulmonary aspergillosis; AA, Aplastic anemia; L-AmB, Liposomal amphotericin B; CBC, Complete blood count; Hb, Hemoglobin; PLT, Platelet count; RBC, Red blood cells; ANC, Absolute neutrophil count; ARC, Absolute reticulocyte count; BM, Bone marrow; rhTPO, Recombinant human thrombopoietin; SC, Subcutaneous; OSH, Outside hospital. Data from OSH were obtained from medical records provided by the patient.

**Figure 2:**
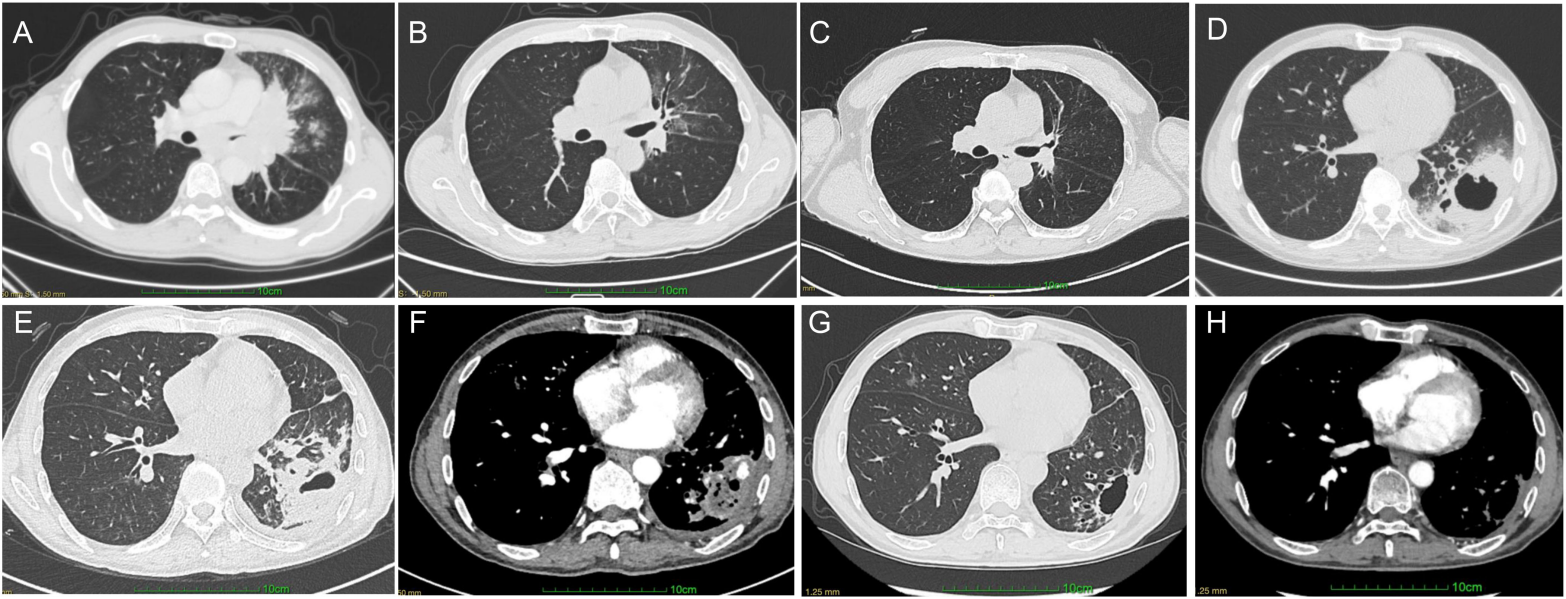
**(A–H)** Serial chest CT images showing evolution of the left hilar tumor and the left lower infectious lesion.

The clinical course is summarized in **Figure 1**. A 58-year-old man with no significant past medical history presented to our hospital in March 2023 with newly diagnosed left-sided squamous NSCLC. Contrast-enhanced chest computed tomography (CT) showed an irregular left perihilar soft-tissue mass involving the left upper lobe and lingula, accompanied by mediastinal and bilateral hilar lymphadenopathy and multiple small nodules in both lungs (**Figure 2A**). A left adrenal lesion was also noted, and metastatic involvement could not be excluded. Overall findings were consistent with cT4N3M1 (stage IV) disease. PD-L1 expression on tumor cells was 20%, and no actionable driver alterations were identified. His Eastern Cooperative Oncology Group (ECOG) performance status was 1. Because he was not a candidate for surgery or radiotherapy, he received pembrolizumab (200 mg every 3 weeks) plus paclitaxel and cisplatin for six cycles, followed by pembrolizumab maintenance monotherapy for a total of 18 cycles (last dose in September 2024). After three cycles, CT showed marked shrinkage of the primary tumor and involved lymph nodes (**Figure 2B**), corresponding to partial response (PR), which remained radiologically stable through September 2024 (**Figure 2C**). Pembrolizumab was withheld after September 2024 due to the onset of severe pulmonary infection in October 2024 and was not resumed because of ongoing infectious complications and subsequent progressive cytopenias.

### 2.2 Recurrent pulmonary infections, development of IPA and antifungal therapy

Approximately one month after the last pembrolizumab dose (October 2024), the patient re-presented with fever and left-sided chest pain. Laboratory testing revealed a leukocyte count of 14.53×10^9^/L, procalcitonin 72.32 ng/mL, and C-reactive protein (CRP) 308 mg/L. Chest CT demonstrated a new, large left lower lobe consolidative lesion with cavitation (**Figure 2D**). Sputum culture yielded *Klebsiella pneumoniae*. Sputum metagenomic next-generation sequencing (mNGS) detected reads from multiple Gram-negative bacilli, predominantly *Raoultella ornithinolytica*. He was treated with intravenous cefoperazone–sulbactam plus oral moxifloxacin, with improvement in inflammatory markers; however, follow-up CT showed radiologic progression of the left lower lobe lesion with increasing cavitation. Bronchoscopy for microbiologic sampling and evaluation of airway involvement was recommended, but the patient declined and requested discharge.

Two months later (December 2024), he was readmitted for cough, sputum production, and hemoptysis. CRP was 19.40 mg/L. Serum (1,3)-β-D-glucan (BDG) and galactomannan (GM) assays and anti-*Aspergillus* IgG were negative. Chest CT demonstrated multifocal patchy opacities and ground-glass changes in both lungs. Compared with the prior scan, the left lower lobe consolidation had partially regressed, but a residual cavity persisted; notably, contrast-enhanced CT (CECT) demonstrated a focal, intensely enhancing intralesional component adjacent to the cavity, suggestive of an angioinvasive change (**Figures 2E, F**). Peripheral blood mNGS identified *Aspergillus niger* (*A. niger*). Based on the clinical course, imaging evolution, and microbiologic evidence, IPA was diagnosed, while concomitant mucormycosis could not be excluded. He received liposomal amphotericin B (L-AmB), followed by oral isavuconazole, and was discharged on oral isavuconazole. A repeat CT in January 2025 demonstrated marked radiologic improvement of the left lower lobe lesion, with resolution of the previously observed focal hyperenhancing intralesional vascular component; however, the left hilar mass and the associated bronchial narrowing had progressed (**Figures 2G, H**).

### 2.3 Progressive cytopenias, bone marrow failure and outcome

**Figure 3:**
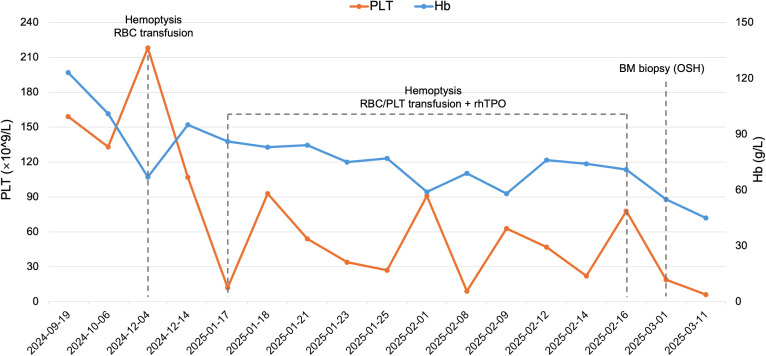
Temporal trends in hemoglobin and platelet count during the clinical course. Hb, Hemoglobin; PLT, Platelet count; RBC, Red blood cells; BM, Bone marrow; rhTPO, Recombinant human thrombopoietin; OSH, Outside hospital. Data from OSH were obtained from medical records provided by the patient.

After discharge in December 2024, the patient continued to experience intermittent cough and sputum production and gradually developed exertional dyspnea; he discontinued isavuconazole prematurely on his own, and progressive pancytopenia subsequently developed. In January 2025, serial complete blood counts showed progressive cytopenias (**Figure 3**). He developed severe anemia and thrombocytopenia (hemoglobin [Hb] 86 g/L; platelet count [PLT] 12 × 10^9^/L) complicated by massive hemoptysis. Bleeding was controlled with hemostatic therapy, red blood cell and platelet transfusions, and subcutaneous recombinant human thrombopoietin, but the hematologic response was transient, and blood counts declined again.

In March 2025, he was evaluated at another hospital, where testing confirmed progressive pancytopenia with reticulocytopenia (absolute neutrophil count [ANC] 1.05 × 10^9^/L; Hb 55 g/L; PLT 19 × 10^9^/L; absolute reticulocyte count [ARC] 0.02 × 10^12^/L). Bone marrow biopsy revealed a markedly hypocellular marrow with residual granulocytic and erythroid precursors but complete absence of megakaryocytes; cytogenetic analysis showed a normal karyotype. These findings were consistent with an AA–like bone marrow failure syndrome. He received systemic corticosteroids and supportive care at the outside institution (detailed dosing information was unavailable). Despite corticosteroid therapy, the most recent complete blood count showed persistent severe cytopenias (ANC 1.54 × 10^9^/L, Hb 45 g/L, PLT 6 × 10^9^/L). He subsequently declined further treatment, requested discharge, and died at home a few days later. Key imaging findings are shown in **Figure 2**, and the overall timeline is summarized in **Figure 1**.

## 3 Discussion

We report a rare case of an advanced squamous NSCLC patient who achieved durable tumor remission after long-term pembrolizumab therapy, but subsequently developed severe irAEs, including IPA and AA-like bone marrow failure. This case highlights the dual role of PD-1 inhibitors: while they can significantly prolong survival and control tumor burden, they also pose the risk of rare but life-threatening complications, which complicate clinical decision-making in patients with advanced malignancy and infection.

### 3.1 Risk and management of IPA during PD-1 inhibitor therapy

In lung cancer patients treated with ICIs, serious infections occur in more than a quarter of cases, with the lung being the most commonly affected site (16). However, monotherapy with PD-1/PD-L1 inhibitors alone confers a relatively modest risk of opportunistic fungal infections, such as IPA (11). Most cases of ICI-associated IPA arise in the setting of irAEs, which require high-dose systemic corticosteroids and other immunosuppressive agents, immune reconstitution inflammatory syndrome (14), or treatment-related cytopenias (17). In this case, the combination of prior platinum-based chemotherapy, local tumor destruction, recurrent bacterial pneumonias, and repeated broad-spectrum antibiotic use, along with prolonged PD-1 blockade, likely created a state of compounded immunosuppression, facilitating the development of IPA.

In patients with hematologic malignancies or profound neutropenia, classic IPA typically presents with pulmonary nodules, halo signs, cavitation, and air-crescent signs on chest CT, which are highly suggestive of the disease (18). However, in non-neutropenic hosts, CT findings are often atypical, and signs like the halo sign are less common, which can easily mimic bacterial pneumonia or tumor progression (19, 20). Published reports of PD-1/PD-L1 inhibitor-associated IPA also mainly involve non-neutropenic patients with additional risk factors such as diabetes, chronic obstructive pulmonary disease (COPD), and long-term corticosteroid use, with CT imaging showing focal consolidation, cavitary lesions, or pneumonia-like features rather than the classic “nodule plus halo sign” pattern (21–23). The primary diagnostic challenge is distinguishing IPA from ICI-related pneumonitis and bacterial pneumonia.

In our case, the atypical CT findings and persistently negative serologic results complicated the diagnosis of IPA. On CECT, a focal hyperenhancing intralesional component adjacent to the cavitary consolidative lesion was observed, raising concern for vascular involvement and an angioinvasive process. This radiologic clue should prompt heightened suspicion for angioinvasive fungal infection, particularly when necrotizing consolidation/cavitation is present and alternative explanations (e.g., bacterial pneumonia or tumor progression) are insufficient. Moreover, *Aspergillus* is rarely recovered from blood cultures in IPA, which limits the diagnostic yield of conventional culture-based testing from peripheral blood (24). In this context, nucleic-acid–based methods, such as mNGS, can detect circulating *Aspergillus* DNA fragments and provide supportive mycological evidence when cultures and serologic biomarkers are negative (25, 26). Accordingly, when bronchoalveolar lavage (BAL) sampling is not feasible, plasma/blood mNGS may serve as a useful adjunct to obtain microbiologic evidence in suspected angioinvasive disease. In our patient, detection of *A. niger* reads in peripheral blood by mNGS—together with compatible imaging, host risk factors, and a marked radiologic response to antifungal therapy—served as strong supportive evidence for IPA, although bronchoscopy with BAL-based testing could not be performed because the patient declined bronchoscopy.

Management of IPA in patients receiving ICIs generally follows guideline-based recommendations, with voriconazole, isavuconazole, posaconazole, or L-AmB as first-line options (27). In cases with suspected or confirmed mucormycosis, high-dose L-AmB is recommended as initial therapy (28). It is crucial to note that even with guideline-concordant antifungal therapy, mortality rates remain high in ICI-treated patients with prior high-dose corticosteroid use or multiple baseline risk factors (22, 29). Accordingly, once IPA is suspected during ICI treatment, immunotherapy should be promptly withheld, systemic antifungal treatment initiated without delay, and microbiologic confirmation sought whenever possible. In our patient, pembrolizumab was initially withheld because of severe active pulmonary infection (before IPA was diagnosed) and was not re-challenged thereafter due to subsequent IPA and progressive cytopenias culminating in AA-like marrow failure. Follow-up imaging showed marked improvement of the left lower lobe lesion, supporting the appropriateness of the antifungal strategy. However, while the infection was controlled, the left hilar tumor continued to progress, and bone marrow failure worsened, leading to an unfavorable outcome. This highlights the therapeutic dilemma of balancing tumor control with the prevention of treatment-related mortality in advanced-stage patients.

### 3.2 ICI-associated AA–like bone marrow failure

Severe hematologic toxicity is a rare but highly lethal category of irAEs, with AA-like bone marrow failure being particularly uncommon (9). Emerging evidence suggests that PD-1/PD-L1 blockade, by removing negative immune regulation, leads to excessive T-cell activation, autoantibody production, and cytokine dysregulation, resulting in a range of Hem-irAEs, including autoimmune hemolytic anemia, immune thrombocytopenia, neutropenia, and bone marrow failure (30). The onset of ICI-related AA is highly variable, occurring from weeks to several months after initiation of therapy, and in some cases, more than a year later (9, 10). Once AA is diagnosed, most patients require permanent discontinuation of ICI therapy, with reported mortality rates approaching 70% (9). Studies show that drug discontinuation or short courses of corticosteroids are often insufficient to reverse marrow failure, whereas some patients achieve hematologic responses with immunosuppressive regimens used for idiopathic AA, such as anti-thymocyte globulin (ATG), cyclosporine A, and eltrombopag (8, 31).

In the differential diagnosis of pancytopenia, infection- and inflammation-related cytopenias were carefully considered, including marrow suppression and/or consumptive thrombocytopenia in the setting of severe bacterial pneumonia, IPA, and bleeding. However, cytopenias continued to worsen despite clinical and radiologic improvement of pulmonary infection, suggesting that infection- or inflammation-related cytopenias alone were unlikely to fully account for the persistent pancytopenia. In addition, antifungal drug–related cytopenia was considered. Although isavuconazole is generally well tolerated, hematologic adverse events, including thrombocytopenia, have been reported in clinical trials and real-world series (32–34). However, in our patient, cytopenias progressed despite discontinuation of isavuconazole, and bone marrow biopsy demonstrated a markedly hypocellular, AA-like phenotype with absent megakaryocytes, making isolated isavuconazole toxicity less likely as the primary etiology. Additionally, although no malignant cells were identified, the evaluation was based on a single-site (unilateral) iliac crest biopsy; given that marrow involvement by malignancy may be focal and that bilateral sampling can reduce false-negative results and increase diagnostic yield (35), occult marrow metastasis cannot be definitively excluded. Importantly, hematologic irAEs have a variable time to onset and may present late during treatment or even after ICI discontinuation; therefore, the several-month interval after the last pembrolizumab dose does not exclude an immune-mediated contribution (36, 37). Our patient exhibited several features consistent with ICI-associated AA-like marrow failure, including late-onset progressive pancytopenia after approximately two years of pembrolizumab therapy, markedly hypocellular marrow with absence of megakaryocytes, a normal karyotype, no evidence of myelodysplastic syndrome, and no new exposure to myelotoxic agents. These findings, along with the temporal relationship and clinicopathologic correlation, support this working diagnosis, and an immune-mediated mechanism was considered in the context of long-term PD-1 blockade. A limitation is the unavailability of high-quality marrow photomicrographs because the biopsy was performed at an outside institution; however, the diagnosis was supported by the detailed pathology report and the concordant clinical course. Prolonged PD-1 blockade may have triggered irreversible immune-mediated marrow injury on a background of reduced hematopoietic reserve related to prior platinum-based chemotherapy and recurrent severe infections. Notably, steroid-refractory courses have been reported in severe hematologic irAEs, and management may require immunosuppression beyond corticosteroids (31, 36). The patient received only corticosteroids and supportive care (with no sustained hematologic response; detailed dosing information was unavailable from outside-hospital records), without timely initiation of standard AA-directed immunosuppressive therapy, which likely contributed to the lack of hematologic recovery. In clinical practice, disproportionate or persistent cytopenias during long-term ICI therapy—particularly progressive pancytopenia with reticulocytopenia and no clear alternative explanation—should prompt early bone marrow evaluation (aspiration/biopsy) with cytogenetic assessment to facilitate prompt drug discontinuation and appropriate intervention.

### 3.3 Limitations

This report has several limitations. First, bronchoscopy with BAL was recommended but declined by the patient, which precluded BAL-based culture and GM testing, as well as other molecular assays, and limited microbiologic confirmation; therefore, peripheral blood mNGS was interpreted as supportive evidence in conjunction with host factors, imaging evolution, and treatment response. Second, part of the hematologic evaluation and treatment were performed at an outside institution, and high-quality marrow photomicrographs and detailed corticosteroid dosing information were unavailable from the records provided. Third, bone marrow assessment was based on a single-site iliac crest biopsy, and focal marrow metastasis cannot be definitively excluded. Finally, as an observational single case, causal inference is inherently limited.

Despite these limitations, this case underscores that in patients who have derived significant oncologic benefit from ICIs, the development of IPA or severe, irreversible Hem-irAEs should prompt a multidisciplinary reassessment, weighing the incremental antitumor benefit of continued immunotherapy against the risk of fatal toxicity. In advanced disease, maximizing ICI exposure is not inherently beneficial; management should prioritize an individualized balance between tumor control, infection risk, and treatment-related mortality.

## 4 Conclusion

This case highlights that while pembrolizumab and other ICIs provide sustained tumor control in advanced NSCLC, they may rarely lead to AA-like bone marrow failure and facilitate opportunistic infections, such as IPA, within a complex immunologic environment. In patients undergoing long-term ICI therapy, close monitoring of blood counts and infection risk is crucial. Unexplained pancytopenia or new/worsening radiologic findings should prompt early bone marrow evaluation and a comprehensive microbiologic work-up. Emerging diagnostic tools, such as mNGS, can provide valuable insights in this context. However, their results should be interpreted within a multidisciplinary framework, alongside conventional investigations, to ensure a proper balance between oncologic benefit and treatment-related toxicity.

## Data Availability

The original contributions presented in the study are included in the article. Further inquiries can be directed to the corresponding author.
